# CD157 and Brain Immune System in (Patho)physiological Conditions: Focus on Brain Plasticity

**DOI:** 10.3389/fimmu.2020.585294

**Published:** 2020-11-10

**Authors:** Olga L. Lopatina, Yulia K. Komleva, Natalia A. Malinovskaya, Yulia A. Panina, Andrey V. Morgun, Alla B. Salmina

**Affiliations:** ^1^ Department of Biochemistry, Medical, Pharmaceutical, and Toxicological Chemistry, Krasnoyarsk State Medical University named after Prof. V.F. Voino-Yasenetsky, Krasnoyarsk, Russia; ^2^ Research Institute of Molecular Medicine and Pathobiochemistry, Krasnoyarsk State Medical University named after Prof. V.F. Voino-Yasenetsky, Krasnoyarsk, Russia; ^3^ Laboratory for Social Brain Studies, Research Institute of Molecular Medicine and Pathobiochemistry, Krasnoyarsk State Medical University named after Prof. V.F. Voino-Yasenetsky, Krasnoyarsk, Russia; ^4^ Department of Biophysics, Siberian Federal University, Krasnoyarsk, Russia

**Keywords:** CD157, immune system, brain plasticity, social brain, brain development

## Abstract

Ectoenzyme and receptor BST-1/CD157 has been considered as a key molecule involved in the regulation of functional activity of cells in various tissues and organs. It is commonly accepted that CD157 catalyzes NAD+ hydrolysis and acts as a component of integrin adhesion receptor complex. Such properties are important for the regulatory role of CD157 in neuronal and glial cells: in addition to recently discovered role in the regulation of emotions, motor functions, and social behavior, CD157 might serve as an important component of innate immune reactions in the central nervous system. Activation of innate immune system in the brain occurs in response to infectious agents as well as in brain injury and neurodegeneration. As an example, in microglial cells, association of CD157 with CD11b/CD18 complex drives reactive gliosis and neuroinflammation evident in brain ischemia, chronic neurodegeneration, and aging. There are various non-substrate ligands of CD157 belonging to the family of extracellular matrix proteins (fibronectin, collagen I, finbrinogen, and laminin) whose activity is required for controlling cell adhesion and migration. Therefore, CD157 could control structural and functional integrity of the blood-brain barrier and barriergenesis. On the other hand, contribution of CD157 to the regulation of brain development is rather possible since in the embryonic brain, CD157 expression is very high, whereas in the adult brain, CD157 is expressed on neural stem cells and, presumably, is involved in the neurogenesis. Besides, CD157 could mediate astrocytes’ action on neural stem and progenitor cells within neurogenic niches. In this review we will summarize how CD157 may affect brain plasticity acting as a molecule at the crossroad of neurogenesis, cerebral angiogenesis, and immune regulation.

## Introduction

NAD+ metabolism is recognized as an important factor contributing to numerous metabolic events and intercellular communications. Several NAD+-consuming enzymes have been discovered in recent 3 decades. Particularly, CD38 and CD157 represent the pair of ectoenzymes involved in the catalytic degradation of NAD+ leading to production of second messengers with pivotal biological effects (i.e., control of Ca2+ release from intracellular stores, regulation of cell movement, etc.) (1–3).

CD157 is well known as bone marrow stromal cell antigen-1 (BST-1) (4), that was first isolated from a bone marrow stromal cells (5), and the BST1 gene was identified by gene cloning as CD157 (6). CD157/BST-1 together with CD38 belong to the NADase/ADP-ribosyl cyclase family, catalyzing the conversion of NAD^+^ and NADP^+^ to cyclic ADP-ribose (cADPR) and nicotinic acid adenine dinucleotide phosphate (NAADP) (4, 7–15). CD157/BST-1 exhibits the same dualism of properties as CD38 like receptor and enzyme activity in leukocytes and ovarian cancer cells (16, 17), bone marrow stromal cells (18), myeloid cells (6, 19, 20), and netrophils and hematopoietic stem cells (14, 18, 21–23).

Interesting, the ADP-ribosyl cyclase activity of CD157 is not so much strong like that one of CD38 including brain. However, it is not clear whether CD157 has other enzyme activities, such as NAD glycohydrolase or NAD base exchange activities. The product of base exchange is nicotinic acid adenine dinucleotide phosphate (NAADP). NAADP also has Ca^2+^ mobilization activity from different Ca^2+^ pools. CD38 initiates calcium mobilization through two products: NAADP and cADPR, but CD157 uses only cADPR pathway (11, 24, 25). Perhaps, sibling rivalry between CD157 and CD38 has become more exiting (26).

From the point of the social brain the role of CD38 in oxytocin (OT) secretion into the brain has been established: CD38 mediates cADPR production, TRMP2 and ERK1/2 activation, Ca^2+^-mobilization, and OT release. Additionally, CD38 is involved in OT release by activating molecular cascades of OT autoregulation (27–30). In contrast, CD157 binds with the serotonin transporter and integrin β and invokes multiple circuits to control anxiety- and depression-like behaviors (24, 31, 32). CD157 plays a role in cADPR-induced OT release, which may not be same to that of CD38. The deficiency of CD157 leads to aberrant behaviors, such as increased anxiety.

Interestingly, despite the important role of CD157 in the immune system (33–35), the CD157/BST1 gene has been identified as a risk factor for neurodegeneration (36), particularly in Parkinson’s disease (PD) (37–48). A new role for CD157 was also found in stem cells when CD157 induces catalysis of cADPR in Paneth cells, which promote self-renewal of stem cells in the intestines in mice on a low-calorie diet (49) and CD157 is responsible for the proliferation of stem and progenitor cells in the lungs (50). The role of CD157 in the regulation of brain activity and plasticity is very intriguing and needs in further evaluation.

## CD157 and Brain Immune Response

CD157 is widely expressed in the brain. CD157 immunoreactivity was detected in the cytoplasm or at the cell surface of many but not all nestin-positive cells in the ventricular and subventricular zones beside the third ventricle (1, 24). In the nervous system, CD157 may play a role in neuronal migration during neural stem cell (NSC) proliferation and neurogenesis. It has been shown that CD157 binds with members of the integrin family (24, 51).

It is also interesting that CD157 is highly expressed in immune cells that are very active in a case of local brain immune response and neuroinflammation. Indeed, microglia cells demonstrate co-expression of CD157 with CD11b and CD18 in experimental Parkinson’s disease in rats (52).

CD11b and CD18 are molecules involved in the regulation of (micro)glial activity being the beta 2-integrin receptor, or complement 3 receptor (Mac-1), or receptor for double-stranded RNA (i.e., of viral origin) (53–55).

CD157 may serve as a part of this complex (56) where its role is in the regulation of leukocyte functional activity. Since activity of Mac-1 as a receptor for extracellular double-stranded RNA (dsRNA), CD157 may be important for sensing dsRNA by glial cells in neuroinflammation as we have suggested before (57). In brain, expression of CD11b/CD18 receptor complex is evident in either astroglial or microglial cells. Stimulation of Mac-1 results in the activation of tall-like receptor-3 (TLR3) and triggers TLR3-independent oxidative inflammatory signaling. Moreover, coordinated activity of Mac-1 and the receptor for advanced glycation end products (RAGE) is involved in development of inflammation, i.e., in neurodegeneration (52, 58). CD157, CD11b, and CD18 co-expression has been detected in microglial cells in experimental Parkinson’s disease in rats (52). It is well-known that activation of TLR3 in blood brain barrier (BBB) cells leads to BBB breakdown, activation of innate immune response and inflammation in the brain affected by degeneration or ischemia (59). Also, TLR3-coupled cell signaling is involved in the disruption of BBB evident in immunologically induced chronic fatigue syndrome (60) when inflammation-induced changes in neuron-glial interactions result in the activation of local immune response and cytokines-driven BBB breakdown. Involvement of viral dsRNA in the pathogenesis of chronic fatigue syndrome that was confirmed in the fatigue model induced by dsRNA poly (I:C) (61), and critical role of CD11b/CD18 complex in the recognition of dsRNA in immune cells (55) provide novel insights on the activity of Mac-1 complex and associated molecules (including CD157). Thus, it is reasonable to speculate that physical and functional association of CD157 with CD11b/CD18 could affect TLR3-dependent and independent signaling, thereby resulting in development of local inflammation caused by the release of PAMPs and DAMPs to the extracellular space, or by the presence of viral dsRNA in the brain tissue.

Therefore, it is not surprising that ATP-mediated purinergic signaling *via* P2X7 receptors induces expression of CD157 in brain microvessel endothelial cells (BMECs) in mice (62), thereby providing a basis for the transmigration of activated peripheral immune cells through the blood-brain barrier into brain tissue. Taking into the consideration the well-established role of CD157 in the regulation of leukocytes movement across the endothelial layer (34), one could propose that integrin-mediated control of the blood-brain barrier integrity might be partly provided by CD157 expressed on brain microvessel endothelial cells. In addition to molecules involved into the control of transendothelial leukocytes migration (CD31, cadherins, JAMs etc.), CD157 expressed by neutrophils and endothelial cells was found to be involved into the regulation of cell adhesion during chemotaxis and transmigration at imflammatory loci (51). This could suggest new clues to the pathogenesis of immune response-associated blood-brain barrier breakdown and progression of neuroinflammation due to excessive migration of peripheral blood leukocytes into the brain tissue at the sites of compromised blood-brain barrier permeability. Moreover, it should be noted, that NAD+ metabolism is of great importance for maintaining metabolic activity of brain microvessel endothelial cells and BBB integrity (63); therefore, catalytic activity of CD157 expressed in endothelial cells might be required for the activation of BMECs seen in brain injury. Recent data on the role of NAD+ depletion in endothelial cells accompanied aging and degeneration further support such assumption (64); however, whether or not it relates to the activation of CD157 remains to be evaluated.

## CD157 and Social Brain

Systemic and local inflammation often associates with behavioral and cognitive deficits (65). Recent data suggest that it might result in prominent alterations in social behavior (66). Moreover, aberrant immune response has been reported in various models of chronic neurodegeneration, autism spectrum disorder (ASD), and schizophrenia (66, 67). As an example, maternal immune activation caused by poly(I:C) drives development of autism-like phenotype in the offspring, demonstrating dependence on purinergic receptors (P2X7) expression in the brain (68). So, the question arises whether CD157 could contribute to the immunity-mediated control of social behavior.

Recently, it was found that the CD157/BST1 gene polymorphisms are associated with some neurological diseases, including ASD (69, 70), which provoked to work with animals with a deletion of the CD157/BST1 gene and to evaluate the possibility of using CD157^−/−^ mice as models of ASD or autistic-like behavior with a social deficit in the absence of motor dysfunctions (especially in childhood and early adolescent).

Social interaction and communication is the most vulnerable behavioral trait in children with ASD (71, 72). Communicative skills are formed in the early period of life and require the use of language as the main tool for the two-way transmission of information. At the same time, mice are social animals with their own communication system with ultrasonic vocalization (USV) in various contexts (73–76). Mouse USV are a whole complex with various qualitative (form, frequency, duration, intensity of sounds, etc.) and quantitative (number of USV produced) (77, 78) characteristics; which, of course, is not a human language.

It was shown that deletion of the *Cd157* gene in mice causes a deviation in the development of the production of ultrasonic vocalization during lactation (32). Young mice, removed from the nest and isolated from the mother, vocalize with a communicative orientation, which correlates with social contacts and research behavior of rodents (79). Neonatal USV can be a guide in understanding remote adult anxiety profiles (78, 80). However, the decrease in vocalization after PND 3, which we observed in CD157^−/−^ mice, can be considered as a delayed violation of communication skills. This may be partially associated with the previously noted autistic (anxious and restless) similar behavior in CD157^−/−^ mice (32).

The structure and organization of the language is actively studied in patients with ASD. Pragmatics is the most “socially motivated” and consistently devalued domain for ASD because it requires an understanding of the language and its correct interpretation during social interactions (72). The language of instruction is tied to mechanical and motor functions, since there is a connection between motor skills and speech function. Atypical motor movements were observed with various phenotypes of ASD (81). Poor vocabulary and its active use in patients with ASD seems to be associated with reduced motivation for communication (82). The registration of USV produced by mouse cubs during social isolation can be distinguished as an index of social motivation of cubs to stimulate parental care and as a marker of early communication deficits in ASD models in mice in anticipation of other, long-term, changes (83, 84). Such scientific studies demonstrate the potential positive effect of introducing oxytocin on increasing motivation for targeted behavior (85) and promoting fundamental psychophysiological functions in the implementation of social behavior, which in turn contributes to social activity (86). Administration of exogenous oxytocin can improve brain function in children with ASD (87). Taking into account all the data we obtained and previously published scientific studies, we assume that the introduction of oxytocin activates the potential flexibility of neuron functions, which does not directly stimulate indirect motivation and, possibly, helps to develop vocabulary and increase vocabulary.

Actually, this is the first study demonstrating the relationship of CD157 with early postnatal development and communicative abilities (which can be restored by introducing exogenous oxytocin). The CD157 gene can be a candidate gene and a risk factor for the development of states of anxiety and social avoidance (social fear). This data can make a great contribution to the study of molecular mechanisms that underlie disturbances in social interactions, in particular, communicative deficits in ASD. However, one study demonstrates that single-nucleotide polymorphisms (SNPs) are rs28532698 and rs4301112 in CD157not predictors of childhood ASD in the Chinese Han population (88), but CD157 sequence variation predicts scores on the Friendship questionnaire (89).

Results of experiments show that CD157^−/−^ young adult male mice displayed anxiety-related behaviors for the novel environment; exhibited anxiety for non-social and/or social novel targets (31, 90). Weak sociability with novel target mice and social avoidance for target males were recover with oxytocin application (31, 32). A new oxytocin analog, lipo-oxytocin-1 (LOT-1), similar to OT, rescued anxiety-like behavior and social avoidance in CD157 knockout mice (91).

**Figure 1:**
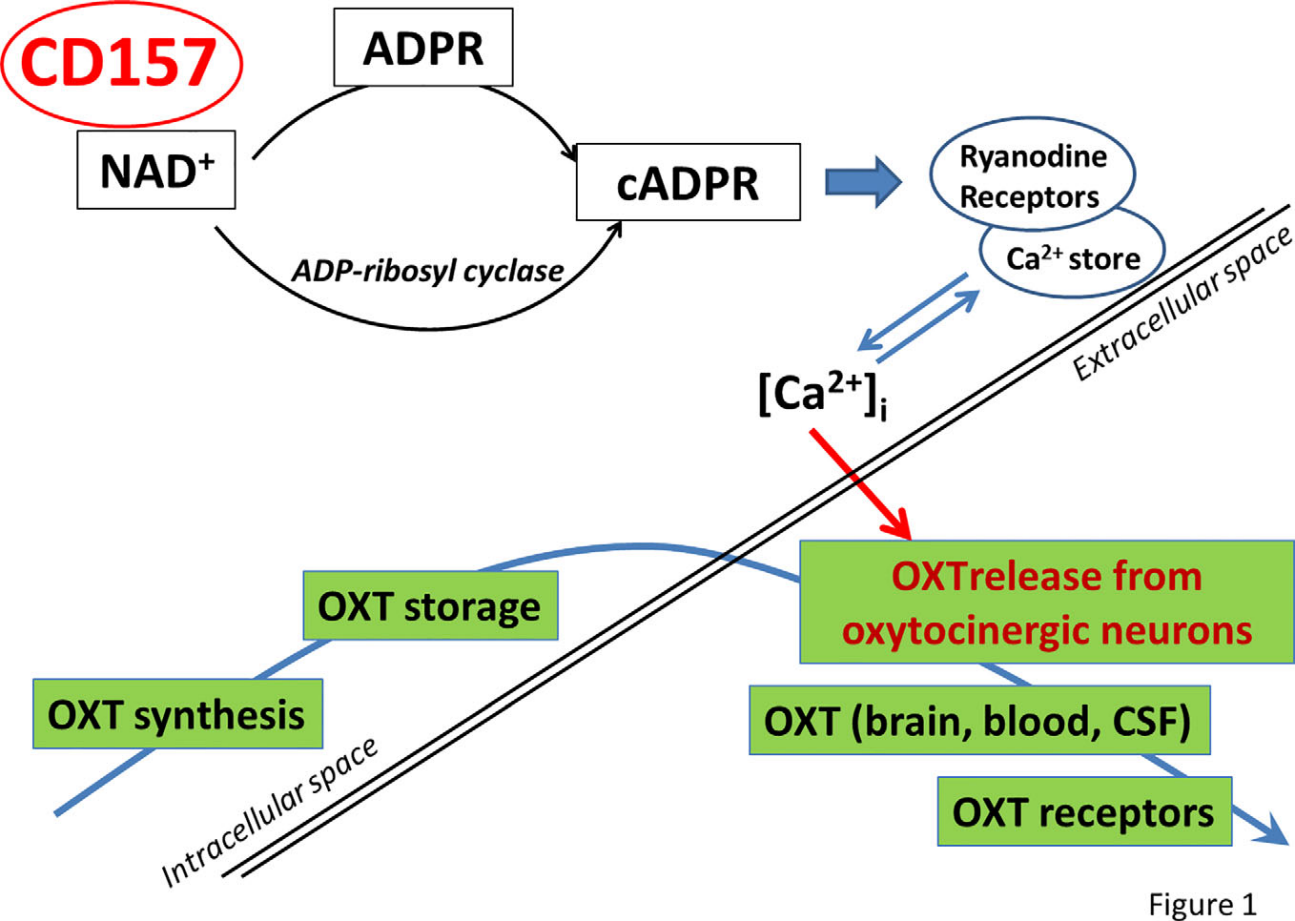
Model illustrating the potential role of CD157 in oxytocin release. CD157 catalyzes NAD+ hydrolysis and synthesis of cADPR, then cADPR acts at Ryanodine receptors expressed in intracellular Ca^2+^ stores to initiate [Ca^2+^]_i_release to cytosole. As a result, OXT is secreted from the cell into the blood and brain tissue. ADPR, ADP-ribose; cADPR, cyclic ADP-ribose; OXT, oxytocin.

In addition, CD157*^−/−^* mice displayed depression-like behaviors and response well for antidepressant treatment (90). Significant differences in the activity of ADP-ribosyl cyclase in the hypothalamus and the pituitary gland between the two genotypes were not observed. Measurement of the oxytocin level in blood plasma showed that the concentration of oxytocin in CD157^−/−^ mice was significantly lower than in mice of the control group (31) (**Figure 1** ). It was interesting to analyze how these behavioral impairments in CD157^−/−^ mice could reflect impairment of the amygdala (31, 92, 93) and what is the role of CD157 in adult neurogenesis. However, there is no direct evidence that CD157 plays a role in neuronal migration during neurogenesis, although CD157 binds integrins in human monocytes and plays a role in neutrophil migration (31, 32).

In sum, CD157 directly or indirectly affects the central axonal release of OXT, which proves the observed change in the oxytocin system (plasma concentration) and the compensatory effect of oxytocin on the behavior of CD157^−/−^ mice. Targeted modulation of CD157 expression in the brain could be considered as an approach to restore behavioral deficits seen in brain disorders associated with impaired social behavior and stress tolerance.

## CD157 and Neuroplasticity

Subgranular zone (SGZ) of hippocampus and subventricular zone (SVZ) are the main areas of the brain where neurogenesis occurs in adulthood due to presence of neurogenic niches with the optimal microenvironment required for maintenance of populations of NSCs, proliferation of neural progenitor cells (NPCs), further differentiation and migration of cells of neuronal and glial lineages (94, 95).

Deletion of the *Cd157* gene is associated with behavioral characteristic of a number of neurodevelopment and neurodegenerative diseases. Violations of socialization, social recognition, and anxiety are associated with learning and memory processes, while autism is associated with impaired synaptogenesis. Moreover, deletion of the *Cd157* gene causes a decrease in the proliferation of neuronal progenitor cells in the SGZ, which can be seen from the significant decrease in the expression of the Nestin marker in the SGZ of the dentate gyrus of CD157^−/−^ mice compared with wild-type C57BL/6 mice (1.43 ± 2.36% and 8.0 ± 2.41%, respectively, P = 0.0039). A tendency toward a decrease in the expression of the marker of neuroblasts (MAP2) in the SGZ of CD157^−/−^ mice (12.64 ± 4.44%) compared with the control group (25.12 ± 5.77%, P = 0.0410). However, no differences were found in the expression of the marker for immature neurons (doublecortin, DCX) in the experimental and control groups, which indicates that CD157 does not control the number of immature neurons.

**Figure 2:**
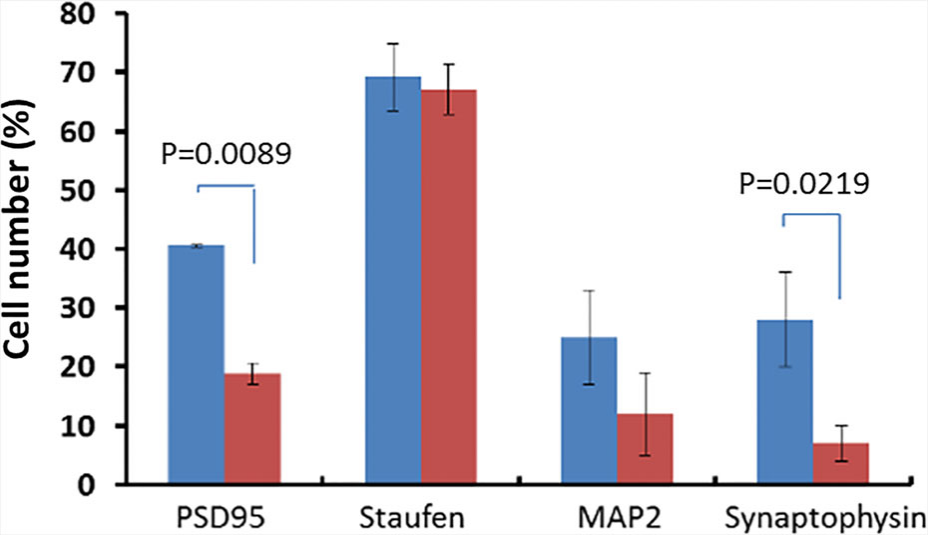
Deficiency of synaptogenesis under deletion of the *Cd157* gene. Expression of synaptogenesis markers (PSD95, Staufen, MAP2, Synaptophysin) in the subgranular zone of the dentate gyrus of the hippocampus in CD157^−/−^ mice (red marker) and in the control group of mice (CD157^+/+^ mice, blue marker). The number of positive cells is presented as a percentage of the total number of cells in the field of view (five visual fields were evaluated). Two-way ANOVA (genotype effect, F (1.32) = 36.26, P = 0.0007) followed by Bonferroni’s *post hoc* test.

There was statistically significant decrease of the expression of the postsynaptic density marker PSD95 in the SGZ of the dentate gyrus in CD157^−/−^ mice (18.79 ± 1.88%) was revealed compared with the control (40.51 ± 0.13%, P = 0.0089, **Figure 2**). We found no difference in Staufen expression (an RNA-binding protein involved in the localization and transport of dendritic mRNA; a marker of neuronal RNA granules) in the dentate gyrus of the hippocampus of CD157^−/−^ mice and wild-type C57BL/6 mice (67.07 ± 4.28% and 69.19 ± 5.67%, respectively, P = 0.7834) (**Figure 2**). But we detected a tendency to a decrease in the expression of MAP2 (associated with protein microtubules) in the same brain area in CD157^−/−^ mice compared to the control group (**Figure 2**). Deletion of the *Cd157* gene is statistically significantly associated with decreased expression of Synaptophysin in the studied brain area (**Figure 2**). These results demonstrate that absence of CD157 expression suppresses synaptogenesis in the hippocampus.

Olfactory bulbs in rodents serve as platform for the integration of newly-formed neurons in neuronal circuits throughout the life being in close functional connection with the SVZ (96). We found significant increase in the expression of CD157 by microglial cells during neurodegeneration in olfactory bulbs (4.12 ± 1.52%) compared with the control group of sham-operated mice (0.72 ± 0.38%, P = 0.027). Also, a statistically significant increase of CD157 expression was recorded in astrocytes expressing GFAP in neurodegeneration group(5.74 ± 1.45%) compared with the sham-operated control (1.03 ± 0.39%, P = 0.05). In S100β+ astrocytes, the expression of CD157 demonstrated tendency to increase (P = 0.086, **Table 1**). Thus, progression of neurodegeneration is accompanied by an increase in the expression of CD157 in microglia cells in rodent olfactory bulbs, thereby suggesting a role of activated microglial cells in the control of newly-formed neurons integration into pre-existing circuits. In microglial cell, similar to immune cells, CD157 is responsible for cytoskeletal rearrangement associated with cell activation and migration and can form a functional complex with the CD11b/CD18, thus contributing to cell adhesion (97). Meta-analysis suggests that the rs11931532 and rs4698412 in CD157, but not rs11724635 might be risk factors for Parkinson’s disease in Asian populations (98).

**Table 1:** The number of CD157+ microglia and astrocytes (%) in olfactory bulbs in control animals and with neurodegeneration.

Groups	Cell type		
	Microglia (MAC-1, CD18/CD11b)	Astrocyte (GFAP+)	Astrocytes(S100β+)
Neurodegeneration (β-amyloid injection)	4.12 ± 1.52	5.74 ± 1.45	2.45 ± 1.48
Control (sham-operated)	0.72 ± 0.38	1.03 ± 0.39	0.46 ± 0.08
*Р*	0.027	0.05	0.086

Animals, n = 5 in every group; slices, n = 5 from every brain; field of view, n = 5 from every slice.

In sum, it is clear that CD157 is involved in the formation of immature neurons and in the proliferation of neuronal cells. In this case CD157 does not affect the number of immature neurons. However, the absence of CD157 negatively affects the processes of synaptogenesis, whereas progression of neurodegeneration is accompanied by CD157 overexpression in brain cells.

Functional association of CD157 and CD200 on stem cells (99) may provide novel role of CD157 in the regulation of stem cells development in neurogenic niches established in a close vicinity to the sites of hyperpermeable BBB (100). Recently, CD157 was confirmed as a marker of tissue-resident vascular endothelial stem cells (VESCs) in large arteries and veins of numerous mouse organs (101), thereby supporting new idea on its role in the immune-controlled regulation of angiogenesis and vascular remodeling (99). It was proposed that CD200+CD157+ endothelial cells (ECs) are self-renewing stem cells contributing to angiogenesis and vasculogenesis by supplying terminally differentiating ECs through a stage of CD200+CD157− endothelial progenitors (102). Moreover, therapeutic application of CD200+CD157+ progenitors aimed to restore angiogenic potential in the affected tissues could be proposed (102). The same approach might be applied for the establishment of new BBB *in vitro* models, particularly those reflecting aberrant barrier integrity evident in neuroinflammation and neurodgeneration (103). In sum, CD157-immunopositive endothelial progenitor cells may display great regenerative potential in tissues, including brain (101); however, nothing is known about this cell pool in the context of brain microvessel development in embryonic or adult stages, and it requires further assessment.

## Conclusion

**Figure 3:**
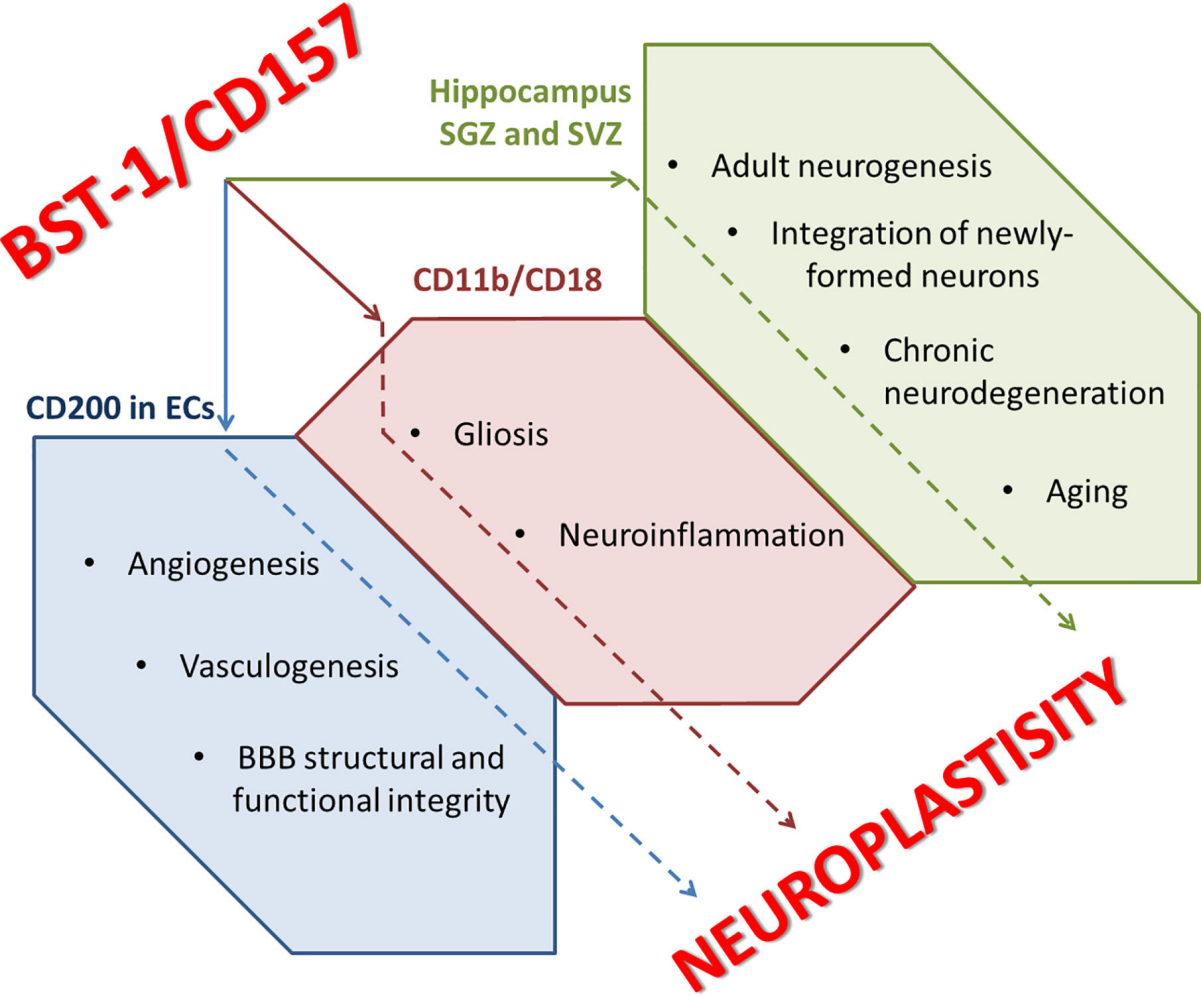
CD157 and neuroplastisity. ECs, endothelial cells; SGZ, subgranular zone of hippocampus; SVZ, subventricular zone of hippocampus.

CD157 is defined as a neuro-entero-immunological regulator. The *Cd157* gene can be a candidate gene and a risk factor for the development of states of anxiety and social avoidance (social fear), and the CD157^−/−^ mice is a relevant model for the study of mental disorders and brain plasticity, including those characteristic of humans. In addition to recently discovered role in the regulation of emotions, motor functions and social behavior, CD157 might serve as an important component of innate immune reactions in the central nervous system. Involvement of CD157 to the regulation of brain development is rather possible since in the embryonic brain where CD157 expression is very high, whereas in the adult brain, CD157 is expressed on NSCs and surrounding glial cells, being, probably, involved in the regulation of adult neurogenesis and integration of newly-formed neurons into pre-existing circuits. Functional association of CD157 with CD11b/CD18 complex may drive reactive gliosis and neuroinflammation evident in brain ischemia, chronic neurodegeneration, and aging. Coupling of CD157 to CD200 in endothelial cells may affect angiogenesis and vasculogenesis, whereas expression of CD157 on mature brain endothelial cells may contribute to controlling BBB structural and functional integrity (**Figure 3**). In sum, CD157 should be recognized as a target molecule for the therapy of brain disorders associated with immune dysfunction, aberrant neuroplasticity, and neuroinflammation.

## Author Contributions

AS and OL conceived of the presented idea. YK, NM, YP, and AM worked on the table and figures. All authors contributed to the article and approved the submitted version.

## Funding

The study is supported by the grant НШ-2547.2020.7 of the President of Russian Federation given to Russian Leading Research Teams.

## Conflict of Interest

The authors declare that the research was conducted in the absence of any commercial or financial relationships that could be construed as a potential conflict of interest.
